# Improving the Bioactivity of Norfloxacin with Tablets Made from Paper

**DOI:** 10.3390/pharmaceutics15020375

**Published:** 2023-01-21

**Authors:** Ayat Abdelkader, Laura Nallbati, Cornelia M. Keck

**Affiliations:** 1 Department of Pharmaceutics and Biopharmaceutics, Philipps-Universität Marburg, Robert-Koch-Str. 4, 35037 Marburg, Germany; 2 Assiut International Center of Nanomedicine, Al-Rajhi Liver Hospital, Assiut University, Assiut 71515, Egypt

**Keywords:** paper, BCS class, dissolution, bioavailability, porous material, drug delivery, oral administration, tablets

## Abstract

(1) Background: Many drugs possess poor bioavailability, and many strategies are available to overcome this issue. In this study, smartFilm technology, i.e., a porous cellulose matrix (paper), in which the active compound can be loaded onto in an amorphous state was utilised for oral administration to improve the solubility and bioactivity of a poorly soluble BSC class IV antibiotic. (2) Methods: Norfloxacin was used as the model drug and loaded into commercially available paper. The resulting norfloxacin-loaded smartFilms were transformed into smartFilm granules via wet granulation and the resulting norfloxacin-loaded smartFilm granules were transformed into norfloxacin-loaded tablets made from paper, i.e., smartFilm tablets. The crystalline state of norfloxacin was investigated, as well as the pharmaceutical properties of the granules and the tablets. The bioactivity of the smartFilm tablets was assessed in vitro and ex vivo to determine the antibacterial activity of norfloxacin. The results were compared to a physical mixture tablet that contained non-loaded paper granules and equal amounts of norfloxacin as a crystalline powder. (3) Results: Norfloxacin-loaded smartFilm granules and norfloxacin-loaded smartFilm tablets contained norfloxacin in an amorphous state, which resulted in an improved and faster release of norfloxacin when compared to the physical mixture tablet. The bioactivity was up to three times higher when compared to the physical mixture tablet. The ex vivo model was demonstrated to be a useful tool that allows for a fast and cost-effective discrimination between “good” and “bad” formulations. It provides realistic physiological conditions and can therefore yield meaningful, additional biopharmaceutical information that cannot be assessed in classical in vitro experiments. (4) Conclusions: smartFilm tablets are a promising, universal, industrially feasible and cost-effective formulation strategy for improved solubility and enhanced bioactivity of poorly soluble drugs.

## 1. Introduction

Many drugs and many new chemical entities possess poor solubility which is associated with poor oral bioavailability [1]. Therefore, strategies that overcome this issue are important to allow for efficient oral drug delivery of these compounds. In recent years, various formulation approaches have been developed for this. Examples include incorporation into micelles, cyclodextrins, self-emulsifying drug delivery systems, microemulsions, liposomes, lipid nanoparticles, solid dispersions, nano-milling or the loading of the active ingredient into porous materials [2,3]. A novel formulation approach is smartFilm technology [4,5]. The smartFilm technology utilizes ordinary paper in which active compounds can be loaded onto in an amorphous state. For this, the active ingredient is dissolved in an appropriate solvent. The resulting solution is then applied to the cellulose-based paper matrix. After drying, the drug is located in the pores of the paper in an amorphous state. This technique is simple and requires no costly equipment [6,7].

The superiority of the smartFilms over other classical (bulk powder suspension) and innovative formulation principles (nanocrystals) has already been demonstrated for dermal applications [8]. For oral drug delivery such a proof-of-concept study has not yet been performed. One reason is the inability to transform smartFilms, i.e., pieces of paper, into a convenient-to-swallow dosage form. This has been overcome by manually transferring paper into paper tablets [6,7]. These tablets were shown to possess sufficient pharmaceutical properties, i.e., they fulfilled the criteria required by the European Pharmacopeia. However, these tablets were produced by manually cutting pieces of paper into small pieces that were then placed into the die of a tabletting machine to be compressed manually. Hence, at that stage, the paper tablets could not be produced on a larger, industrial scale. A technology that cannot be produced on a large, industrial scale cannot be transferred into real world products. Therefore, a method that allows for the production of paper tablets on a larger scale was developed [9]. This method was a three-step process. In the first step the paper was milled. The milled paper was transformed into paper granules via a wet granulation process. The paper granules then served as an intermediate product for the production of paper tablets.

Paper granules without additional excipients can be produced but will not result in paper tablets with sufficient pharmaceutical properties. Such paper granules possess a high elasticity and can therefore jump out of the die during the compression process. The elastic properties can be decreased, and plastic deformation can be improved by using sucrose as a binder during the wet granulation process. This results in paper granules with optimal flowability and compressibility. The compression of these paper granules results then in paper tablets with optimal pharmaceutical properties [9].

The above-mentioned results were obtained from non-loaded paper. Hence, with these drug-free tablets it was not possible to prove that smartFilms can indeed improve the bioactivity of poorly soluble drugs after oral application. Therefore, in a following study, curcumin-loaded smartFilms were produced and transferred into smartFilm granules. The curcumin-loaded smartFilm granules were transferred into curcumin-loaded smartFilm tablets and characterised regarding their pharmaceutical properties. In addition, the oral bioavailability, i.e., intestinal permeability, was assessed in an ex vivo porcine intestinal model. The results demonstrated that wet granulation did not impair the amorphous state of the curcumin and resulted in curcumin-loaded smartFilm granules with good flowability which could be transferred into curcumin-loaded smartFilm tablets with good pharmaceutical properties. The ex vivo bioavailability was superior when compared to physical mixture paper tablets. Therefore, data currently provide evidence that smartFilm tablets are an industrially feasible approach for the formulation of poorly soluble drugs [10]. 

In the previously mentioned study, the smartFilm tablets were already shown to increase the dissolution rate and the intestinal permeability of curcumin but no data were available for other active ingredients. A study that proves that smartFilms and smartFilm tablets can really improve the pharmacological efficacy in the gastrointestinal tract is also not available. These data are considered to be essential to provide further evidence for the usefulness of the smartFilm technology and to prove that the formulation principle is universal, i.e., can be used to enhance the solubility and bioactivity for different types of poorly soluble active ingredients. Such a proof-of-concept study was therefore performed here. 

Norfloxacin is a BSC class IV drug with poor solubility and poor intestinal permeability [11]. It is a broadband antibiotic that belongs to the class of fluoroquinolone antibiotics. It affects Gram-positive and Gram-negative bacteria and thus can be used for the treatment of various diseases [12]. In this study, according to the previously established protocols [9,10], norfloxacin-loaded smartFilms were prepared and transformed into smartFilm granules from which smartFilm tablets were prepared. The crystalline state of norfloxacin within the granules and the tablets was determined and the pharmaceutical properties of the granules and tablets were assessed according to the European Pharmacopeia. The antibacterial activity of the norfloxacin-loaded tablets was assessed in vitro and in an ex vivo model. The results obtained were compared to physical mixture tablets that contained non-loaded paper granules and identical amounts of norfloxacin as a crystalline powder.

## 2. Materials and Methods

### 2.1. Materials

Norfloxacin, 95%, was purchased from abcr GmbH (Karlsruhe, Germany). Commercially available, cellulose-based paper (Soft & Sicher, dm-drogerie markt GmbH + Co. KG, Karlsruhe, Germany) was utilised as the paper matrix. Sucrose, peptone, beef extract, sodium chloride and potassium chloride were acquired from Carl Roth GmbH + Co. KG (Karlsruhe, Germany). Agar was purchased from Sigma-Aldrich Pty Ltd. (Darmstadt, Germany). Magnesium sulfate heptahydrate, magnesium chloride hexahydrate, calcium chloride dihydrate, sodium hydrogen carbonate, di-potassium hydrogen phosphate and di-sodium hydrogen phosphate were acquired from VWR International GmbH (Darmstadt, Germany). *Aliivibrio fischeri* (*A. fischeri*) bacterial strain (ATCC 7744/ NCMB 1281) was obtained from Dr. G. Schuchardt^®^ (Göttingen, Germany). Purified water was freshly obtained from a PURELAB Flex 2 (ELGA LabWater, Veolia Water Technologies GmbH, Celle, Germany). 

### 2.2. Methods

#### 2.2.1. Production and Characterization of Norfloxacin-Loaded smartFilms and smartFilm Granules

Norfloxacin-loaded smartFilms and smartFilm granules were prepared as described previously [9,10], with slight modifications. In the first step, norfloxacin was dissolved in a mixture of acetone and ethanol (ratio of 1:1) to produce a solution that contained 2.5 mg/mL norfloxacin. The obtained norfloxacin solution (0.5 mL) was loaded onto paper sheets (5 × 5 cm^2^) with an individual mass of approximately 200 mg by using an automatic micropipette. The paper sheets were left to dry, then the process was repeated several times to prepare 20 mg norfloxacin-loaded paper sheets (i.e., smartFilms). The dried norfloxacin-loaded smartFilms were then used to produce norfloxacin-loaded smartFilm granules. For this, the smartFilms were dry milled using a knife mill (Moulinex DP8108, Groupe SEB Deutschland GmbH, Frankfurt, Germany) for 1 min. The milled smartFilms were mixed with sucrose to obtain milled smartFilms that contained 20% (w/w) sucrose. Purified water was sprayed on top of the milled smartFilm/sucrose mixture in order to dissolve the sucrose and to increase the density of the blend. The mixture was further ground for 1 min and then transferred to a plastic sieve. On the sieve, the mixture was further wet with purified water under shaking at 300 rpm (universal shaker SM-30 control, Edmund Bühler GmbH, Bodelshausen, Germany) for a period of 3–8 min. The resulting wet, norfloxacin-loaded smartFilm granules were dried in an oven for 30 min at 120 °C (UN 30, Memmert GmbH + Co. KG, Schwabach, Germany). Afterwards, the granules were sieved (mesh size 2.8 mm, Retsch GmbH, Haan, Germany) to obtain a size fraction ≤ 2.8 mm. The smartFilm granules were characterised regarding their size and pharmaceutical characteristics (i.e., bulk density, tapped density, Hausner ratio, Carr Index, and angle of repose). The crystalline state of norfloxacin was determined by X-ray diffractometry. Details of the methods used are given below.

##### Particle Size

The particle size of the smartFilm granules was determined from macroscopic images of the granules. For this, ten representative images that contained approximately 250–300 granules were taken by using a Canon IXUS 190 digital camera (Canon Europe Ltd., Uxbridge, UK). The images obtained were subjected to digital image analysis by using ImageJ software (National Institutes of Health, Bethesda, MD, USA), as described previously [13]. In the first step, the images were color-adjusted, and threshold analysis was performed to mark the granules individually. Then, the Feret diameter of each granule was evaluated by an automated software algorithm (Supplementary Materials, Table S1). The results were then used to calculate the number based median particle size diameters d(*n*) 0.10, d(*n*) 0.50, d(*n*) 0.90, d(*n*) 0.95 and d(*n*) 0.99 with JASP software, version 0.16.2 [14].

#### Pharmaceutical Characteristics

The pharmaceutical characteristics of the norfloxacin-loaded smartFilm granules that were assessed included bulk density, tapped density, Hausner ratio, Carr index and the angle of repose.

**Determination of bulk and tapped density**

The bulk and tapped density of norfloxacin-loaded smartFilm granules were determined according to the test method 2.9.34. of the European Pharmacopeia by using a mechanical tapping device (tap density tester TD200, Pharma Test Apparatebau AG, Hainburg, Germany) [15]. An amount of 10 g of the granules was placed into a 250 mL measuring cylinder. The initial volume and the final volume, after performing 10, 500 and 1250 taps on the same granule sample, were noted, and used to calculate the bulk and tapped density, respectively. 

**Determination of Hausner ratio and Carr index**

From the obtained density results, the Hausner ratio and the Carr index, i.e., flowability parameters, were calculated using the following equations [16]:

(1) $$Hausner\;ratio=\frac{\rho_{tapped}}{\rho_{bulk}}\;$$

(2) $$Carr\;index=100\left(\frac{\rho_{tapped-}\rho_{bulk}}{\rho_{tapped}}\right)\;$$

where ρ_*tapped*_ is the tapped density and ρ_*bulk*_ is the bulk density.

**Angle of repose**

The angle of repose was assessed according to the test method 2.9.36. of the European Pharmacopeia [15]. An amount of 20 g of the granules was placed in the funnel of a flowability tester (Emmeram Karg Industrietechnik, Krailling, Germany). Then, the granules were gently stirred to pass through the funnel and piled up on a fixed base to form a heap. The height of the granule heap was measured, and the angle of repose (α) was calculated using the following equation:

(3) $$\tan\alpha =\frac{h}{0.5\;d_{b}}\;$$

where *h* is the height of the heap and *d_b_* is the diameter of the base.

#### Crystalline State of Norfloxacin

X-ray diffraction (XRD) patterns were studied to assess the crystalline state of norfloxacin loaded within the smartFilm granules and the smartFilm tablets. For this, an X-ray powder diffractometer (X’Pert Pro MDP, PANalytical/Philipps BV, Netherlands) was used to record the X-ray diffraction patterns from smartFilm tablets from the corresponding references (norfloxacin raw bulk material, physical mixture of paper and bulk norfloxacin, norfloxacin-loaded smartFilm granules and unloaded paper granules with 20% sucrose content). The instrument was equipped with CuK α radiation (λ = 1.7903 Å) and operated at a voltage of 40 kV and a current of 35 mA at room temperature. Samples were scanned from 2θ = 10° to 2θ = 55° with a step of 0.03°/s. 

#### 2.2.2. Production and Characterization of Norfloxacin-Loaded smartFilm Tablets

The norfloxacin-loaded smartFilm granules were compressed into flat-faced bevel-edged tablets by using a single punch tablet press (EK0, Korsch GmbH, Berlin, Germany) equipped with a 10 mm flat-faced punch (Ritter Pharma-Technik GmbH, Stapelfeld, Germany). The compression force was 30 kN. Physical mixture tablets that contained similar amounts of norfloxacin as raw powder and unloaded paper granules were also obtained via this process. The crystalline state of norfloxacin within the smartFilm tablets was determined with X-ray diffractometry (cf. Crystalline State of Norfloxacin) and the pharmaceutical characteristics of the produced smartFilm tablets were evaluated according to the tests described in the European Pharmacopeia [15]. Details of the methods used are given below.

##### Pharmaceutical Characteristics

**Tablet thickness and mass uniformity**

Ten tablets were randomly selected, and their thickness was determined with a IP67 ABS digital caliper (Mitutoyo, Kanagawa, Japan). The mass uniformity was evaluated according to the test method 2.9.5. of the European Pharmacopeia [15]. The test was conducted using 20 randomly selected tablets. The tablets were weighed, then the average weight was calculated and compared to the weight of each individual tablet to determine the percentage deviation. Afterwards, the results obtained were compared to the European Pharmacopeia limits.

**Friability**

The friability of 20 randomly selected tablets was evaluated according to test method 2.9.7. of the European Pharmacopeia. The tablets were dedusted and accurately weighed, then placed in the drum of a friability tester PTF 10ER (Pharma Test Apparatebau AG, Hainburg, Germany) [15], and the drum was set to rotate 100 times. Subsequently, the tablets were dedusted, accurately weighed and the percentage weight loss was calculated using the following equation:

(4) $$\%\;weight\;loss=100\left(\frac{W_{1}-W_{2}}{W_{1}}\right)\;$$

where *W*_1_ is the weight of the tablets before the test and *W*_2_ is the weight of the tablets after the test.

**Resistance to crushing**

The force required to break down a tablet under compression, i.e., the crushing strength or hardness, was assessed according to the test method 2.9.8. of the European Pharmacopeia [15]. Tablets (*n* = 10) were individually placed in a horizontal plane between the jaws of a hardness tester PTB 311E (Pharma Test Apparatebau AG, Hainburg, Germany), then the result was expressed in Newtons (N) as a mean value of the forces measured. 

**Disintegration**

Disintegration of tablets (*n* = 6) was evaluated in water according to the test method 2.9.1. of the European Pharmacopeia [15]. Tablets were individually positioned in the cavities of a disintegration tester PTZ-S (Pharma Test Apparatebau AG, Hainburg, Germany) operating at a temperature of 37 °C ± 2 °C, and the time required for full tablet disintegration was noted.

**Content uniformity**

The content uniformity within the smartFilm tablets was investigated according to test method 2.9.6. of the European Pharmacopeia [15]. Norfloxacin-loaded smartFilm tablets (*n* = 10) were individually immersed in phosphate-buffered saline (PBS, pH 6.8) under stirring until complete disintegration of each tablet. Samples were withdrawn and filtered using a syringe filter with a pore size of 0.22 µm (Otto E. Kobe KG, Marburg, Germany). Afterwards, the amount of norfloxacin within each tablet was determined spectrophotometrically at the predetermined λ_max_ of norfloxacin (320 nm) using UV–Vis spectroscopy (Multiskan™ GO, Thermo Fischer Scientific, Waltham, MA, USA) and a preconstructed calibration curve (10–20 mg/L). 

**Dissolution**

Dissolution studies were conducted according to the test method 2.9.3. of the European Pharmacopeia [15] using PBS solution (pH 6.8) as a dissolution medium and a paddle apparatus PTWS 120D (Pharmatest, Apparatebau AG, Hainburg, Germany). Norfloxacin-loaded smartFilm tablets (*n* = 6) were individually dropped into the apparatus vessels which were filled with 900 mL PBS solution (pH 6.8). The paddle speed was set at 100 rpm and the temperature was maintained at 37 ± 0.5 °C. Samples (10 mL) were withdrawn after 5, 10, 20, 40, 60, 90, 120, 180, 240, 360 and 480 min and replaced with an equal volume (10 mL) of the fresh dissolution medium. All samples were filtered using a syringe filter (0.22 µm) and were then divided in two equal aliquots. One aliquot was analysed regarding the amount of dissolved norfloxacin (UV–Vis spectroscopy, Multiskan™ GO, Thermo Fischer Scientific, Waltham, MA, USA, λ = 320 nm, preconstructed calibration curve from 10–20 mg/L) and the second aliquot was used for the determination of the in vitro antibacterial activity (cf. In Vitro Antibacterial Activity). A similar experiment was carried out by using a physical mixture of tablets that was produced from a blend of unloaded paper granules with 20% sucrose content and the same content of norfloxacin powder. 

#### 2.2.3. Evaluation of Antibacterial Activity

The antibacterial activity of the formulations was tested against *A. fischeri*. *A. fischeri* is a Gram-negative, rod-shaped bacterium with bioluminescent properties [17]. The luminescence intensity is directly proportional to the metabolic activity of the bacteria and can therefore be used as a sensitive indicator to determine the toxicity, i.e., antimicrobial activity, of chemical compounds [18]. In this study, the changes in bioluminescence upon treatment with the different formulations were determined in vitro and ex vivo. For both setups, the bacteria were prepared following a previously established protocol [19]. *A. fischeri* cultures were grown overnight in photobacterium medium (28.13 g NaCl, 0.77 g KCl, 1.2 g CaCl_2_·2 H_2_O, 3.6 g MgCl_2_·6 H_2_O, 0.0825 g NaHCO_3_, 2.625 g MgSO_4_·7 H_2_O, 10 g peptone, 10 g beef extract per 1 L distilled water) under shaking at 24 °C (Incubator Hood TH 15, Edmund Bühler, Bodelshausen, Germany) until an optical density of 0.6 at 600 nm (OD600) was achieved (UVmini-1240 Spectrophotometer, Shimadzu™ Europa GmbH, Duisburg, Germany). 

##### In Vitro Antibacterial Activity

The in vitro antibacterial activity was determined by bioluminescence assay, i.e., by assessing the bioluminescence inhibition (%) of *A. fischeri* over time [18]. For this, 100 µL of sample was added into a well of a 96-well flat bottom white polystyrene microtiter plate. Then, the well was inoculated with 100 µL of *A. fischeri* culture that was freshly diluted (1:1) with photobacterium medium prior to use. The resulting luminescence intensity was measured with an integration time of 1000 ms per well after 12 h by using an Infinite 200Pro microplate reader equipped with an OD2 filter (Tecan Group Ltd., Männedorf, Switzerland). The experiment was performed in triplicate. An amount of 100 µL photobacterium growth medium with 100 µL *A. fischeri* culture was used as a positive control and 200 µL photobacterium growth medium without *A. fischeri* culture was used as a negative control. The bioluminescence inhibition (% *BL* inhibition) was calculated using the following equation:

(5) $$\%\;BL\;\mathrm{inhibition}=100-\left(\frac{100\cdot {\left(BL_{sample}-BL_{negative\;control\;}\right)}_{\;}}{BL_{positive\;control}-BL_{negative\;control}}\right)\;$$

where *BL_sample_* is the measured bioluminescence intensity of the respective sample, *BL_negative control_* is the measured bioluminescence intensity of the negative control and *BL_positive control_* is the measured bioluminescence intensity of the positive control.

##### Ex Vivo Antibacterial Activity

The ex vivo antibacterial activity was determined from porcine intestines [10]. Fresh intact porcine gastrointestinal tracts were acquired from a local slaughterhouse and were used for the experiments within 2 h after slaughter. From the intestines obtained, small sections (approximately 15 cm in length) of intestinal tissue (i.e., 15–20 cm away from the pylorus) were isolated, dissected longitudinally and spread as a sheet. The intestinal sheets were gently wiped without affecting the villi structure and the pre-existing mucus. Subsequently, 2 mL of *A. fischeri* culture was taken and separately centrifugated at 1000 rpm for 10 min at room temperature (MIKRO 120 centrifuge, Andreas Hettich GmbH & Co. KG, Tuttlingen, Germany). The resulting pellets were individually resuspended in 30 μL fresh medium and 60 μL of the obtained bacterial suspension was applied to each intestinal test area. The *A. fischeri*-infected intestinal tissues were incubated at room temperature for two hours to allow the bacteria to grow. 

During the incubation time, the norfloxacin-loaded smartFilm tablets and the physical mixture tablets were subjected to a pre-digestion procedure to mimic the digestion and changes in the tablets after oral administration [10]. For this, one tablet of each formulation was separately immersed in 500 mL phosphate-buffered saline (pH 6.8, 37 °C) and stirred for 10 min at 300 rpm (universal shaker SM-30 control, Edmund Bühler GmbH, Bodelshausen, Germany). After this time, aliquots (100 µL) were withdrawn from each formulation and applied onto the *A. fischeri*-infected intestinal tissues (i.e., 2 h post infection). One infected area on each intestinal tissue was left untreated and served as control. Swabs from the surface of all intestinal areas were taken using a sterilised cotton swab at 0, 2, 4, 6, 8, 12 and 24 h post-treatment. The bacterial swabs were separately cultured on artificial seawater agar plates using the spread plate technique and incubated at room temperature overnight [20]. The experiments were performed in triplicate and the luminescence intensity of each plate was determined after 18 h.

**Figure 1:**
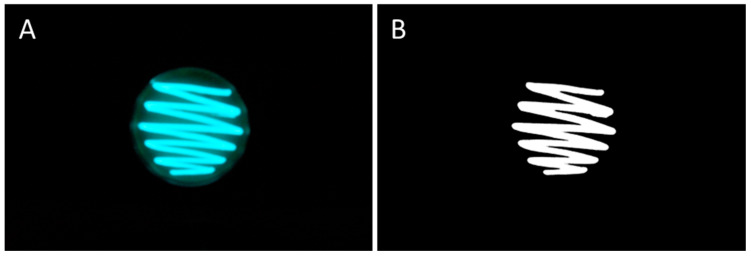
(**A**): Macroscopic image of an agar plate in the dark cultured with *A. fischeri* swabs after 18 h. (**B**): Macroscopic image after the automated threshold algorithm (Supplementary Materials, Table S2) that subtracted the background of the image. The remaining white pixels correspond to the bioluminescence intensity of *A. fischeri*.

For this, each plate was photographed in the dark (Figure 1A) using a Nikon D7200 Digital SLR camera equipped with an 18–300 mm lens (Nikon Europe B.V., Amsterdam, The Netherlands). The luminescence intensity from each plate was then determined semi-quantitatively by subjecting the images to digital image analysis described previously [13,21,22,23,24], with slight modifications. ImageJ software, version 1.53 k, was used for the analysis [25,26].

The first step was an automated color adjustment (Supplementary Materials, Table S2) of the image that allowed for the separation between class I (foreground) and class II (background) pixels. The foreground pixels correspond to the luminescence of the bacteria and the background pixels correspond to the surrounding media. The second step subtracted the class II pixels from the class I pixels (Supplementary Materials, Table S2). The remaining pixels in the resulting image (Figure 1B) represent the bioluminescence intensity of the bacteria after the respective treatment. In this way, the ex vivo luminescence intensity could be assessed as mean grey value ((MGV)/px) from each image. The results are presented as mean values of the bioluminescence intensity ± standard deviation from the different replicates of each formulation and from the different time points of incubation.

#### 2.2.4. Statistical Analysis

Descriptive statistics and statistical assessment of differences between the mean values were performed using JASP software version 0.16.2 (Universiteit van Amsterdam, Amsterdam, The Netherlands) [14]. Normal distribution was checked with the Shapiro–Wilk test and variance homogeneity was checked with Levene’s test. Subsequently, the data were subjected to one-way analysis of variance (ANOVA, normally distributed data) or the Kruskal–Wallis tests (non-parametric data). Adequate post hoc tests (Tukey’s, Games–Howell and Dunnett and Dunn [27]) were performed to compare the mean values with each other. In some cases, for a more detailed comparison of the data, *t*-tests for pairwise comparison were also performed. The Pearson correlation coefficient is a measure of linear correlation between two sets of data. It was determined in between the in vitro dissolution data, the in vitro antibacterial activity data and the ex vivo antibacterial activity data [28]. Data are represented as mean ± standard deviation, unless otherwise stated. Differences between means were considered statistically significant if the *p* value was <0.05. 

## 3. Results

### 3.1. Production and Characterization of Norfloxacin-Loaded smartFilm Granules

**Figure 2:**
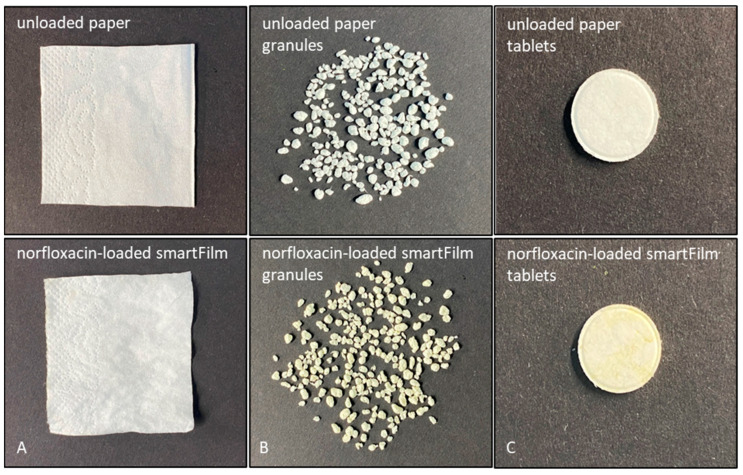
Macroscopic images of (**A**): unloaded paper (upper) and paper loaded with norfloxacin (smartFilms, lower), (**B**): unloaded paper granules (upper) and norfloxacin-loaded smartFilm granules (lower) (**C**): unloaded paper tablets (upper) and norfloxacin-loaded smartFilm tablets (lower).

Norfloxacin smartFilm granules were successfully produced from the norfloxacin-loaded smartFilms (Figure 2A,B). The granules were further characterised regarding size, their pharmaceutical characteristics and regarding the crystalline state of norfloxacin within the granules.

#### 3.1.1. Particle Size

**Figure 3:**
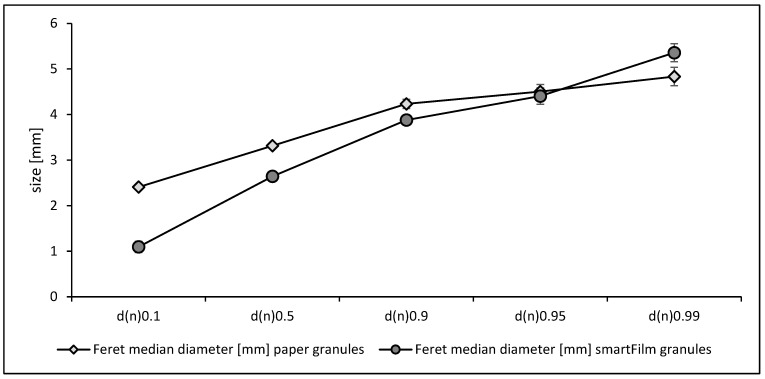
Particle size (numeric median diameters) of norfloxacin-loaded smartFilm granules in comparison to unloaded paper granules.

The Feret diameter of the unladed paper granules was 3.3 ± 0.9 mm and was 2.6 ± 1.0 mm for the norfloxacin-loaded smartFilm granules. The numeric size distribution showed that the d(*n*) 0.1, d(*n*) 0.5 and d(*n*) 0.9 values were significantly lower for the smartFilm granules. The d(*n*) 0.95 values were similar but the d(*n*) 0.99 value was higher for the smartFilm granules (Figure 3). This means the norfloxacin granules possessed a slightly smaller mean particle size and a slightly broader size distribution than the unloaded paper granules. The smaller size of the smartFilm granules can be explained by an increased density of the cellulose matrix that is caused by loading the drug into the pores of the paper [10]. The trend towards some larger particles can also be explained by the increased density of the drug-loaded smartFilm granules, i.e., due to the reduced pore size, the uptake of the binding agent into the pores is reduced, which then reduces the granulation efficiency of the binder. 

#### 3.1.2. Pharmaceutical Characteristics

The pharmaceutical characteristics of the norfloxacin-loaded smartFilm granules, i.e., bulk density, tapped density, Hausner ratio, Carr index and angle of repose, were determined in the next step (Table 1). The differences in bulk and tapped density were small, which resulted in a Hausner ratio of <1.19, a Carr index of <15 and an angle of repose of <40. The data indicate good to moderate flowability [29]. Hence, industrial compression of the granules into tablets was considered to be possible without complications. The data also support the theory that the smaller particle size of the smartFilm granules is due to their higher density when compared to unloaded paper granules. Both, the bulk and the tapped density increased in the paper granules upon loading the paper with norfloxacin when compared to the unloaded paper granules. As expected, the resulting smaller particle size and the slightly larger size distribution changed the particle interaction forces, which was observed in a slightly reduced flowability of the smartFilm powder (i.e., increased Hausner ratio and Carr index), when compared to the unloaded paper granules (Table 1). Similar effects were also found when curcumin was loaded into paper [10]. Based on these results, it can therefore be concluded that the loading of active compounds into paper resulted in an increased density of the paper. The transfer of drug-loaded paper into granules resulted in granules with a smaller size, broader size distribution and lower flowability. 

#### 3.1.3. Crystalline State of Norfloxacin

**Figure 4:**
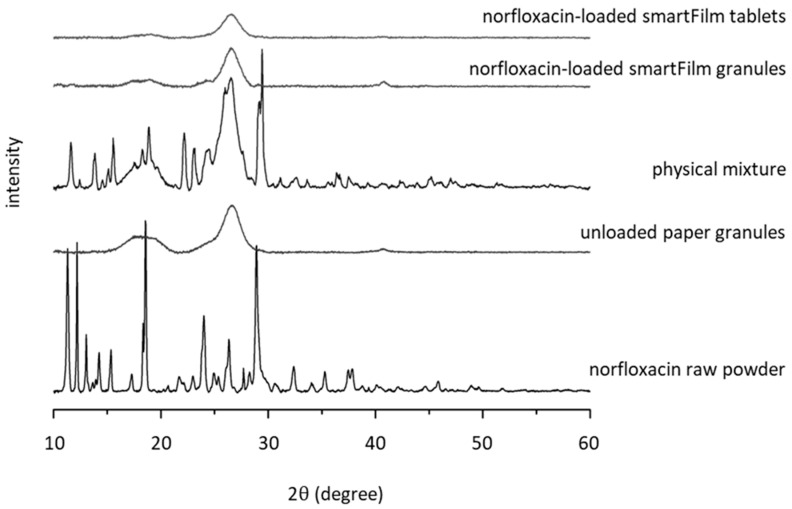
X-ray diffraction patterns of norfloxacin-loaded smartFilm granules and smartFilm tablets in comparison to norfloxacin raw powder, unloaded paper granules and the physical mixture of norfloxacin and paper.

The smartFilm technology aims to increase the solubility of poorly soluble drugs by transferring and maintaining the drug in its amorphous state. The production of smartFilm granules via wet granulation runs the risk that the drug is (partially) dissolved during the granulation process and might re-crystallize upon drying. The formed crystals might trigger further crystallization of amorphous norfloxacin. X-ray analysis, however, showed that the granulation process did not alter the crystalline state of norfloxacin (Figure 4). 

The diffractogram of norfloxacin raw powder revealed the typical reflexes of norfloxacin in a crystalline state. The physical mixture of norfloxacin raw powder and unloaded paper granules also showed these reflexes. In contrast, no reflexes were found for the norfloxacin-loaded smartFilm granules that contained similar amounts of norfloxacin as the physical mixture. This therefore indicates that norfloxacin is loaded into the smartFilm granules in an amorphous state.

Based on these data, it was concluded that the norfloxacin-loaded smartFilm granules are suitable intermediate products for the production of norfloxacin-loaded smartFilm tablets. Therefore, in the next step of the study, the norfloxacin-loaded smartFilm granules were compressed into tablets and their resulting properties were analysed.

### 3.2. Production and Characterization of Norfloxacin-Loaded smartFilm Tablets

#### 3.2.1. Crystalline State of Norfloxacin

The compression of the norfloxacin-loaded smartFilm granules into smartFilm tablets resulted in smooth tablets with a shiny surface (Figure 2C) and did not alter the crystalline state of the norfloxacin (Figure 4). No reflexes and no changes in the diffractogram in comparison to the norfloxacin-loaded smartFilm granules were observed for the smartFilm tablets, indicating that norfloxacin was loaded into the smartFilm tablets in an amorphous state.

#### 3.2.2. Pharmaceutical Characteristics

The pharmaceutical properties (e.g., thickness of tablet, mass uniformity, content uniformity, friability, etc.) of the norfloxacin-loaded smartFilm tablets are summarised in Table 2 and show that the norfloxacin-loaded smartFilm tablets fulfil the requirements according to the European Pharmacopeia. The average mass uniformity value was 2.9 ± 1.2% and no tablet out of the 20 weighed tablets had an individual mass that differed by more than 7.5% from the average mass (tablet mass was in the range of 80–250 mg). The average content uniformity value was 97.5 ± 1.5%, and no tablet out of the ten examined tablets exhibited a content value that differed by more than 15%. The disintegration time was <15 min, the friability was <1% and the crushing strength value was about 111 ± 12 N. Thus, the disintegration time of the tablets was sufficiently fast and their mechanical strength was sufficiently high [29].

A comparison of the pharmaceutical properties of the norfloxacin-loaded smartFilm tablets to the properties of the tablets obtained with non-loaded paper granules shows that the properties of the tablets are altered upon loading with norfloxacin (Table 2). Upon loading, the thickness of the tablets, the friability and the disintegration time increased, while the mass uniformity decreased. The crushing strength was not altered. The changes in mass uniformity can be linked to the reduced flowability of the smartFilm granules, which results in a less constant flow of the granules into the die of the tablet press and thus leads to stronger variation in the tablet mass. The increased thickness reduced friability and increased disintegration time can be linked to the higher density of the smartFilm granules.

The higher density was due to the loading of the drug into to the pores of the paper, which also reduces the porosity of the paper matrix. Hence, the loaded smartFilm granules contain less air within the pores of the paper; therefore, the compression at similar compression forces yielded thicker tablets. The friability, crushing strength and disintegration are influenced by different parameters that can interfere with each other. In the case of the smartFilm granules loaded with norfloxacin, the increased density when compared to the unloaded paper granules seems to be responsible for the increased disintegration time, i.e., the reduced pore size seems to decrease the wettability of the tablet and thus increases its disintegration time. The increased density can be considered to increase the hardness of the material, which then seems to reduce the friability of the tablets. However, such an increase in hardness should also lead to an increase in crushing strength. An increase in crushing strength for smartFilm tables was indeed observed in a recent study when curcumin was loaded into paper [10]. However, such an increase was not seen in this set of data. A possible explanation is a less efficient plastic deformation of the norfloxacin-loaded smartFilm granules during compression due to the lower porosity of the loaded smartFilm granules and the lower predicted compressibility due the increased Carr index. For the production of tablets with optimal and tailor-made pharmaceutical properties, more systematic research is needed to investigate and understand the interplay between granule properties, drug loading and resulting tablet properties in detail. Nonetheless, the data from this study clearly confirm that it is possible to produce norfloxacin-loaded smartFilm tablets that fulfil the requirements according to the European Pharmacopeia. The resulting biopharmaceutical properties of the smartFilm tablets (release kinetics and antibacterial activity) were therefore determined in the next step of the study.

#### 3.2.3. Dissolution Profile

**Figure 5:**
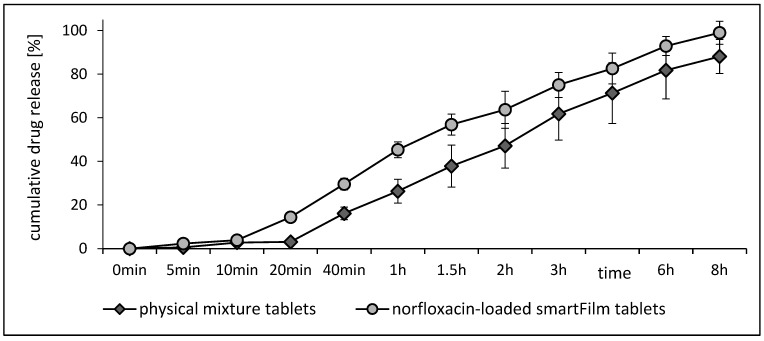
Cumulative drug release of norfloxacin from norfloxacin-loaded smartFilm tablets in comparison to physical mixture tablets.

The dissolution profile of the smartFilm tablets showed a biphasic release profile, i.e., a very slow release at early time points (<10 min) and a faster release after ≥20 min. A biphasic release profile was also obtained for the physical mixture tablets. However, the onset of the fast release was delayed, and the total amount of dissolved active material was lower when compared to the smartFilm tablets (Figure 5). The amount of released norfloxacin was significantly higher for the smartFilm tablets after 5 min, 10 min, 20 min, 40 min, 1 h and 1.5 h. 

The difference was most pronounced between 10 and 40 min (about a 2-fold difference) and declined and became non-significant with higher dissolution times (2 h–6 h). After 8 h, the amount of released norfloxacin from the smartFilm tablets was about 12% higher when compared to the released norfloxacin from the physical mixture (Student’s *t*-test, one-tailed, *p* < 0.05). From the obtained data, it can be concluded that the smartFilm tablets show an enhanced release of norfloxacin when compared to the physical mixture. Hence, in comparison to the physical mixture, the data suggest a higher bioactivity of the smartFilm tablets. This assumption was therefore tested in the next part of the study by determining the antibacterial activity of the smartFilm tablets in vitro and in an ex vivo model. 

#### 3.2.4. Determination of Antibacterial Activity

##### In Vitro Antibacterial Activity

**Figure 6:**
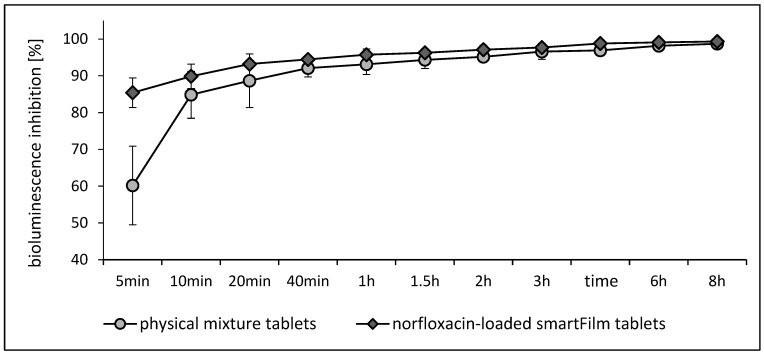
Antibacterial activity (bioluminescence inhibition of *A. fischeri*) of norfloxacin from smartFilm tablets in comparison to physical mixture tablets.

The in vitro antibacterial activity of norfloxacin from norfloxacin-loaded smartFilm tablets was determined and compared with the in vitro antibacterial activity of norfloxacin physical mixture tablets (Figure 6). The tests were performed with *A. fischeri* that possesses bioluminescent properties [17]. The bioluminescence extinguishes if the treatment is toxic to the bacterium. Therefore, the bioluminescence inhibition can be used to compare the antibacterial activity of different formulations [18]. A bioluminescence inhibition of 100% means that 100% of the bacteria were deactivated by the treatment.

Treatment with the norfloxacin-loaded smartFilm tablets caused a fast increase in the bioluminescence inhibition. After 5 min, 85% of the bacteria showed an extinguished luminescence and after 10 min, 90% of the bacteria were affected by the treatment, i.e., the decimal reduction time can be estimated to be 10 min. A proportion of 99% of the bacteria luminescence was inactivated after 4 h (Figure 6). The physical mixture tablets possessed a less efficient antibacterial activity. After 5 min, about 60% of the bacteria were affected by the treatment and after 10 min, 85% of the bacteria were affected. The decimal reduction time was about 30 min and 99% of the bacteria luminescence was depleted after 8 h. Hence, based on the decimal reduction time and the time needed to affect 99% of the bacteria, the antibacterial activity of norfloxacin from smartFilm tablets was about 2- to 3-fold higher when compared to the physical mixture tablets. The differences in antibacterial activity between smartFilm tablets and physical mixture tablets were expected and can be related to the faster release of norfloxacin from the smartFilm tablets (cf. Figure 5).

##### Ex Vivo Antibacterial Activity

**Figure 7:**
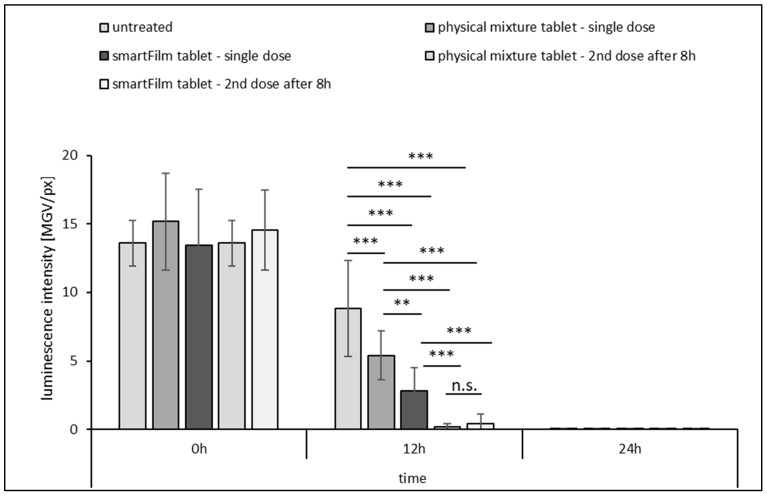
Ex vivo antibacterial activity from infected intestinal tissue (bioluminescence of *A. fischeri*). Non-treated vs. treated with norfloxacin-loaded smartFilm tablets in comparison to physical mixture tablets applied as a single dose or in second (booster) dose setup after 8 h (explanations cf. text). ** *p* < 0.01, *** *p* < 0.001, n.s.: not significant.

The ex vivo antibacterial activity was assessed in porcine intestines that were infected with *A. fischeri*. The formulations were applied onto the infected intestinal tissue and the number of active bacteria was determined by remaining bioluminescence at different time points. The first experiment was performed for a period of 24 h, where samples were taken after 0 h, 12 h and 24 h. The experiment was performed to roughly estimate the time frame for the ex vivo method that can yield reliable and discriminative results between different samples and application setups. Samples applied were the pre-digested smartFilm tablets and the pre-digested physical mixture tablets. The two different formulations were applied on the intestinal tissue either once (application time = 0 h) or twice (application time = 0 h and 8 h), with the untreated infected intestine sections serving as the control (Figure 7).

The data show that the number of active bacteria was similar for all infected intestines tested at the beginning of the experiment. After 12 h, significant differences were found between the untreated and differently treated tissues and after 24 h, the luminescence was extinguished in all samples. The results show that the model yields reproducible results (similar numbers of bacteria at the beginning from different intestinal tissues) and that it remains sensitive within a time frame of at least 12 h (i.e., significant differences and changes can be found in between different formulations and application setups). After this time, all bacteria were inactive, independent of whether the tissues were treated with antibacterial formulations or not (Figure 7). 

The data also show that the smartFilm tablets exhibit a significantly higher antibacterial activity than the physical mixture tablets when the formulations were applied once. The decrease in bacterial luminescence after 12 h was about one-third for the physical mixture tablets (−39 ± 13%) and about two-thirds (−68 ± 17%) for the smartFilm tablets. Hence, an approximate 2-fold increase in the antibacterial activity of the smartFilm tablets was found when compared to the physical mixture tablets. With this observation, the data are in line with the results obtained in the in vitro bioluminescence assay, which showed a similar trend for the decimal reduction time and the time needed to deactivate 99% of the bacteria (cf. Figure 6). The less efficient and slower deactivation of the bacteria in the ex vivo model when compared to the data from the in vitro model might be caused by the lower amounts of norfloxacin being available after the pre-digestion step and/or a slower diffusion of the released norfloxacin within the tissue. An additional possibility is the metabolism of the norfloxacin via intestinal enzymes or other interactions that might (partially) inactivate it.

The half-life of norfloxacin was about 3 h, but might be prolonged with decreased enzyme activity [30]. As the ex vivo antibacterial activity was assessed after 12 h, which is much later than the half time of norfloxacin, it was reasonable to hypothesize that the norfloxacin might have been partially deactivated during this time. This would mean that the antibacterial effect of the two formulations that were applied only once was not optimal. In addition, it would also mean that the antibacterial effect could be enhanced by applying a second dose. This theory is substantiated by the results obtained when the formulations were applied twice, which resulted in a decrease in luminescence of ≥95% for both formulations (Figure 7). 

**Figure 8:**
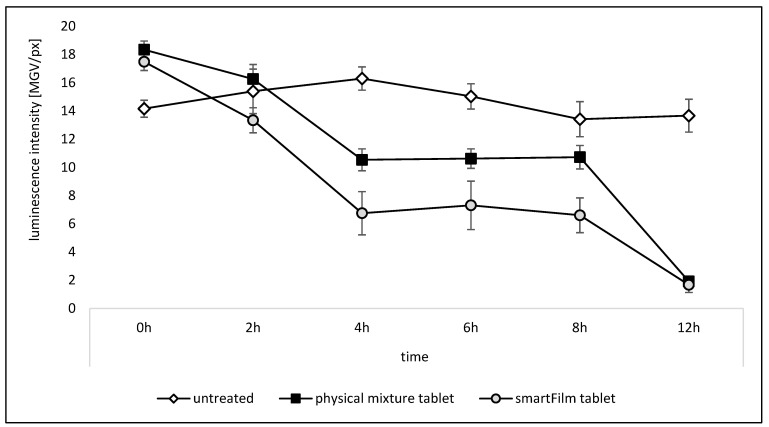
Ex vivo antibacterial activity (luminescence of *A. fischeri*) after treatment with norfloxacin-loaded smartFilm tablets in comparison to physical mixture tablets (explanations cf. text).

In the next step, we aimed to investigate the differences between the two formulations in more detail, i.e., at different time points within the predetermined time frame of the ex vivo model. For this, the formulations were applied on the intestines, and samples were taken at 2 h, 4 h, 6 h, 8 h and 12 h after application. After 8 h, a second dose, i.e., a booster dose, was applied and final samples were taken after 12 h. Untreated tissues served as controls (Figure 8). At the beginning, slightly fewer active bacteria were located on the non-treated intestinal sections. After 2 h, the differences were cancelled out between untreated controls and the physical mixture tablets, i.e., all sections had a similar luminescence intensity. The tissue sections treated with the smartFilm tablets resulted in a lower luminescence intensity (Mann–Whitney test, *p* < 0.05). The results therefore indicate that the smartFilm tablets acted faster than the physical mixture tablets. After 4 h, all treated intestine sections showed a lower luminescence intensity than the untreated intestine sections and the effect was significant for both formulations. The smartFilm tablets resulted in significantly less luminescence intensity than the physical mixture tablets. After 6 h and 8 h, the differences between the formulations became smaller, but remained significant. For both formulations, after 4 h, a plateau in antibacterial activity was reached. Hence, in between 4 h and 8 h no further decrease in the antibacterial activity was observed. After 12 h, and after application of the second dose, the activity of the bacteria declined to almost zero, with no significant differences between the smartFilm tablets and the physical mixture tablets (Figure 8).

The obtained data are in line with the expectations, i.e., both the physical mixture tablets and the smartFilm tablets were expected to possess antibacterial properties. The smartFilm tablets, due to the better solubility of norfloxacin, were expected to possess a more pronounced antibacterial activity. In addition, due to the faster release of norfloxacin from the smartFilm tablets, it was expected that the antibacterial effect would be detectable at earlier time points when compared to the physical mixture with slower release kinetics for norfloxacin. The results proved the faster onset of antibacterial activity for the smartFilm tablets and their more pronounced antibacterial activity. However, they also showed that the antibacterial effect reaches a plateau after 4 h. The effect is considered to be related to the short half-life of the norfloxacin, which seems to become (partially) inactive after this time. Only the application of a 2nd dose (booster dose) could further reduce the bacterial activity. After 12 h, and after the application of the booster dose, the antibacterial activity was similar for the smartFilm tablets and the physical mixture tablets. This indicates that the smartFilm tablets are especially useful if a fast antibacterial action is required. For longer, multiple dose treatments, the use of a classical formulation approach seems to be sufficient. From an ecological point of view, it might be most efficient to apply the initial dose with a smartFilm tablet to yield a fast onset of the antibacterial activity and to apply the classical formulation as maintenance dose. In this way, the treatment would be highly efficient and cost-effective (intellectual property protected formulations are more costly) at the same time. Future research is now needed to investigate this in more detail. 

**Figure 9:**
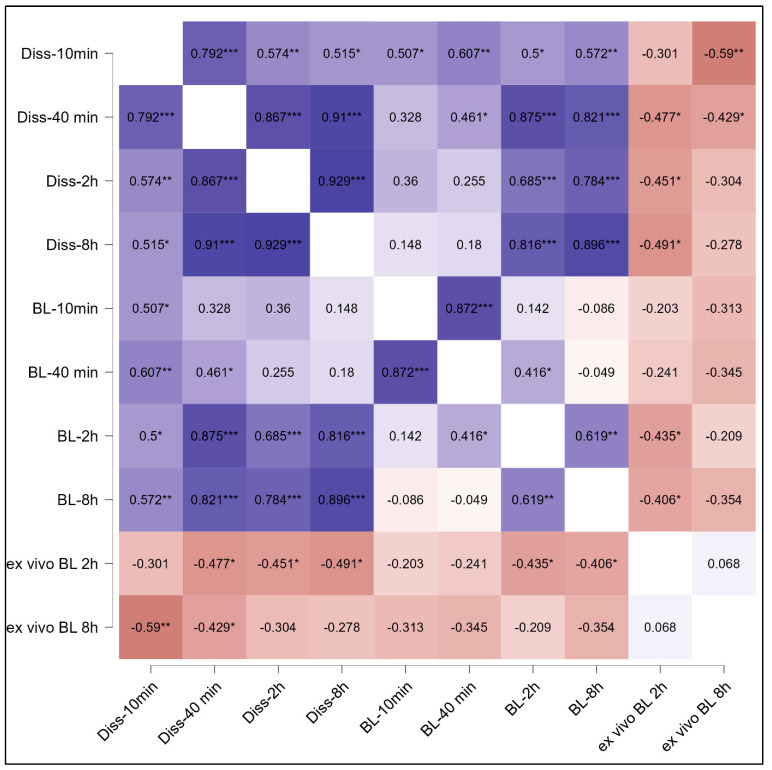
Heatmap of Pearson correlation coefficients (selected time points) that assess the relationship between the in vitro dissolution data (Diss), the in vitro bioluminescence inhibition (BL) and the ex vivo antibacterial activity (ex vivo BL) data for the physical mixture and the norfloxacin-loaded smartFilm tablets at different time points. * *p* < 0.05, ** *p* < 0.01, *** *p* < 0.001.

A correlation between the in vitro data and the ex vivo data can be typically analysed to judge the predictability of a model [31]. However, norfloxacin is a BSC IV drug [11]; therefore, a good in vitro in vivo (ex vivo) correlation cannot be expected [31]. The correlation of the data from this study confirmed this (Figure 9, Supplementary Materials, Figure S1). Figure 9 represents some selected time points indicates that the correlation coefficient between the different dissolution (Diss) time points is not constant. This is due to the anomalous release (non-Fickian) diffusion, i.e., biphasic release, of norfloxacin (cf. Figure 5). Hence, only the time points from the slow release in the beginning show a good correlation to each other but result in low correlation coefficients with higher dissolution times that are associated with a faster drug release, and vice versa. A similar effect can be seen for the in vitro bioluminescence data (BL), i.e., the BL data from early time points correlate well with early time dissolution data but result in poor correlation with higher dissolution time points. Higher BL time points correlate well with higher dissolution times (Figure 9). 

The ex vivo data were acquired with pre-digested formulations that were applied to the intestine after 10 min pre-digestion (cf. Ex Vivo Antibacterial Activity). Hence, in contrast to the correlation between dissolution data and bioluminescence inhibition, where the norfloxacin was released throughout the entire experiment, in this part of the study, only one drug concentration, corresponding to a dissolution time of 10 min, was used. Therefore, the best correlation between dissolution, bioluminescence inhibition and ex vivo antibacterial activity should be found when the ex vivo antibacterial activity is assessed after 10 min. However, this correlation is not possible, because the ex vivo antibacterial activity was assessed after 2 h, 4 h, 6 h, 8 h and 12 h post application of the formulations onto the intestinal tissue. Hence, the early time points that can be expected to correlate well with the early dissolution data were not acquired. Such an early determination of the ex vivo antibacterial activity is also not feasible, because an ex vivo model should estimate the biological effect in a realistic setup and environment [32]. 

Therefore, based on the chemical nature of norfloxacin and the related considerations, a strong and linear correlation between the in vitro data and the ex vivo data is not possible. However, the model should still provide a clear trend that shows the effect of a formulation in relation to an in vitro property. In this case, it is expected that the higher amount of drug released causes a more pronounced antibacterial activity. This would result in a positive correlation between dissolution data and in vitro bioluminescence inhibition and would result in a negative corelation between the in vitro data and the ex vivo antibacterial activity, which was assessed as the remaining luminescence of the bacteria after the treatment, i.e., a low remaining ex vivo luminescence of the treatment indicates good ex vivo antibacterial activity. This expected negative correlation between in vitro and ex vivo data was found for all time points (Figure 9, Supplementary Materials, Figure S1). 

Based on the correlation data, it is not possible to estimate the real accuracy of the model because many parameters are not linear. Despite this, the data clearly demonstrate that the ex vivo model is able to judge the antibacterial activity from different formulations. It enables a rough estimation of differences in pharmacokinetic profiles and provides a clear link to the in vitro data, thus rendering the ex vivo model a useful tool that allows for a fast and cost-effective discrimination between “good” and “bad” formulations. 

In contrast to the in vitro test methods, the ex vivo model bears manifold parameters that provide a close link to real physiological conditions (i.e., mucus, pH, active enzymes, etc.). Therefore, its use can be considered to yield meaningful, additional biopharmaceutical information that cannot be assessed from classical in vitro experiments. The model can be considered to be especially useful in early formulation development, because at this stage it is often not necessary to have highly accurate results. In most cases, it is sufficient to obtain a rough estimate of whether a formulation is effective or not and whether changes in the formulation and/or treatment improve the performance or not. In this way, effective formulations can be developed in a cost-efficient and time-saving way. 

The data acquired here provide evidence that smartFilm tablets can be utilised as a simple, efficient formulation strategy for the improved solubility and enhanced bioactivity of norfloxacin. Nevertheless, future work should now focus on providing a detailed picture about the underlaying mechanisms for this. For this, systematic studies that investigate, e.g., the interactions between the paper matrix and the active pharmaceutical ingredients (API) and the influence of the paper matrix and production parameters on the chemical stability of the API and the pharmaceutical and biopharmaceutical properties, are required. These mechanistic data will then be the base for a sound development of effective smartFilm tablets that allow for an improved bio-efficacy of poorly water-soluble API.

## 4. Conclusions

Norfloxacin-loaded smartFilm tablets were successfully produced in this study. The tablets maintained the norfloxacin in an amorphous state and possessed sufficient pharmaceutical properties (resistance to crushing, mass uniformity, content uniformity, friability, and disintegration time). The tablets showed a biphasic release profile of the norfloxacin. The dissolution rate was up to three times higher when compared to the physical mixture tablet. This also resulted in an up to three-fold increase in in vitro antibacterial activity. The ex vivo antibacterial activity showed a similar trend, i.e., a higher bioactivity of norfloxacin in the smartFilm tablets when compared to the physical mixture tablet. The more physiological environment of the ex vivo model could provide a more detailed understanding on the effects (formulation aspects and application aspects) that influence the bioactivity of the norfloxacin. The results provide further evidence that smartFilm tablets are a universal, industrially feasible formulation strategy for the improved solubility and enhanced bioactivity of poorly soluble drugs.

## Figures and Tables

**Table 1:** Pharmaceutical properties of norfloxacin-loaded smartFilm granules in comparison to unloaded paper granules.

	Bulk Density (g/cm^3^)	Tapped Density (g/cm^3^)	Hausner Ratio	Carr Index	Angle of Repose
Paper granules *	0.14 ± 0.00 *	0.15 ± 0.00 *	1.13 ± 0.04 *	11.7 ± 3.6 *	31° ± 0 *
smartFilm granules	0.15 ± 0.00	0.18 ± 0.00	1.17 ± 0.00	14.9 ± 0.0	31° ± 0

* data from [9].

**Table 2:** Pharmaceutical properties of norfloxacin-loaded smartFilm tablets in comparison to unloaded paper tablets.

	Thickness (mm)	Mass Uni-formity (%)	Content Uni-formity (%)	Friability (%)	Resistance to Crushing (N)	Disintegration
Paper tablets *	1.7 ± 0.04 *	1.8 ± 1.6 *	n.a.	0.23 *	112.8 ±18.6 * min.: 84.2 max.: 129.5	All tablets disintegrated within 5 min *
smartFilm tablets	2.3 ± 0.05	2.9 ± 1.8	97.5 ± 1.5 max: 99.3	0.07	110.7 ± 12.2 min.: 82.5 max.: 125.9	All tablets disintegrated within 15 min

* data from [9].

## Data Availability

Not applicable.

## Supplementary Materials

The following supporting information can be downloaded at: https://www.mdpi.com/article/10.3390/pharmaceutics15020375/s1, Figure S1: Heatmap of Pearson correlation coefficients that assess the relationship between the in vitro dissolution data (Diss), the invitro bioluminescence inhibition (BL) and the ex vivo antibacterial activity (ex vivo BL) data for the physical mixture and the norfloxacin-loaded smartFilm tablets at different time points; Table S1: Macro used for the determination of the smartFilm granules particle size; Table S2: Macro used for the automated threshold to subtract the background from the luminescence of the *A. fischeri.*
