# Supplementary material for: Improving the Bioactivity of Norfloxacin with Tablets Made from Paper
Source: Pharmaceutics. 2023 Jan 21;15(2):375. doi: 10.3390/pharmaceutics15020375 (PMC9959448; doi:10.3390/pharmaceutics15020375)

pharmaceutics

Article

Improving the Bioactivity of Norfloxacin with Tablets Made from Paper

Ayat Abdelkader [1,2], Laura Nallbati [1] and Cornelia M. Keck [1,*]

[1] Department of Pharmaceutics and Biopharmaceutics, Philipps-Universität Marburg, Robert-Koch-Str. 4, 35037 Marburg, Germany
[2] Assiut International Center of Nanomedicine, Al-Rajhi Liver Hospital, Assiut University, Assiut 71515, Egypt
* Correspondence: cornelia.keck@pharmazie.uni-marburg.de; Tel.: +49-6421-282-5881

Supplementary Materials:

Table S1: Macro used for the determination of the smartFilm granules particle size.

```
run("8-bit");
setAutoThreshold("Default");
//run("Threshold...");
//setThreshold(0, 180);
setOption("BlackBackground", false);
run("Convert to Mask");
run("Analyze Particles...", "size=0.09-Infinity show=Masks display");
```

Table S2: Macro used for the automated threshold to subtract the background from the luminescence of the *A. fischeri*.

```
// Color Thresholder 1.53k
// Autogenerated macro, single images only!
min=newArray(3);
max=newArray(3);
filter=newArray(3);
a=getTitle();
run("RGB Stack");
run("Convert Stack to Images");
selectWindow("Red");
rename("0");
selectWindow("Green");
rename("1");
selectWindow("Blue");
rename("2");
min[0]=98;
max[0]=255;
filter[0]="pass";
```

```
min[1]=186;
max[1]=255;
filter[1]="pass";
min[2]=199;
max[2]=255;
filter[2]="pass";
for (i=0;i<3;i++){
   selectWindow(""+i);
   setThreshold(min[i], max[i]);
   run("Convert to Mask");
   if (filter[i]=="stop")    run("Invert");
}
imageCalculator("AND create", "0","1");
imageCalculator("AND create", "Result of 0","2");
for (i=0;i<3;i++){
   selectWindow(""+i);
   close();
}
selectWindow("Result of 0");
close();
selectWindow("Result of Result of 0");
rename(a);
// Colour Thresholding------------
run("Invert");min[1]=75;
max[1]=255;
filter[1]="pass";
min[2]=0;
max[2]=0;
filter[2]="stop";
for (i=0;i<3;i++){
   selectWindow(""+i);
   setThreshold(min[i], max[i]);
   run("Convert to Mask");
   if (filter[i]=="stop")    run("Invert");
}
imageCalculator("AND create", "0","1");
imageCalculator("AND create", "Result of 0","2");
for (i=0;i<3;i++){
   selectWindow(""+i);
   close();
}
selectWindow("Result of 0");
```

```
close();
selectWindow("Result of Result of 0");
rename(a);
// Colour Thresholding-------------
run("Invert");
```

Figure S1: Heatmap of Pearson correlation coefficients that assess the relationship between the in vitro dissolution data (Diss), the invitro bioluminescence inhibition (BL) and the ex vivo antibacterial activity (ex vivo BL) data for the physical mixture and the norfloxacin-loaded smartFilm tablets at different time points. * p < .05, ** p < .01, *** p < .001.

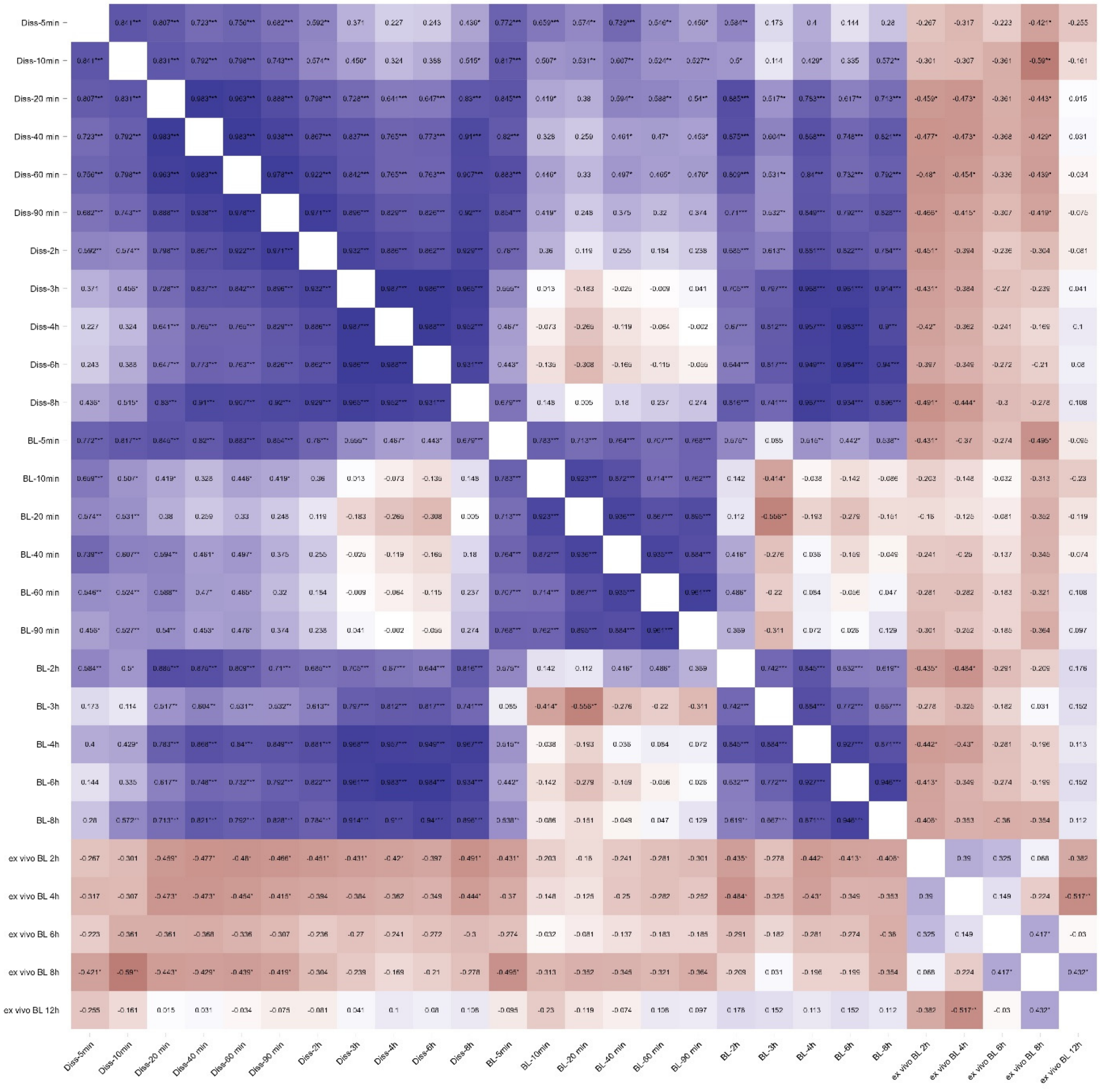

Supplement: Supplementary file 1 [file pharmaceutics-15-00375-s001.zip › pharmaceutics-2033843-supplementary.pdf]
